# Prasugrel: Reassessing Its Role in Contemporary Antiplatelet Therapy

**DOI:** 10.1155/cdr/5538781

**Published:** 2026-06-27

**Authors:** Yusof Kamisah, Hamat Hamdi Che Hassan

**Affiliations:** ^1^ Department of Pharmacology, Faculty of Medicine, Universiti Kebangsaan Malaysia, Kuala Lumpur, Malaysia, ukm.my; ^2^ Cardiovascular and Respiratory Research Group, Universiti Kebangsaan Malaysia, Bandar Baru Bangi, Selangor, Malaysia, ukm.my; ^3^ Department of Medicine, Faculty of Medicine, Universiti Kebangsaan Malaysia, Kuala Lumpur, Malaysia, ukm.my

## Abstract

Managing acute coronary syndrome (ACS) and atherosclerotic cardiovascular disease remains clinically challenging, requiring a careful balance between ischemic protection and bleeding risk. Antiplatelet therapy is central to the management, with aspirin serving as the cornerstone; however, selection of the second antiplatelet agent in dual antiplatelet therapy (DAPT) remains complex. Clopidogrel, a purinergic receptor subtype Y, G protein–coupled, 12 (P2Y_12_) inhibitor, is widely used but is limited by substantial interindividual variability in antiplatelet response, particularly in populations with a high prevalence of CYP2C19 loss‐of‐function polymorphisms. These limitations prompted the development of more potent P2Y_12_ inhibitors, including prasugrel and ticagrelor, which provide faster and more consistent platelet inhibition. However, clinical experience—especially in Asian populations—has highlighted that a “one‐size‐fits‐all” dosing strategy may not be appropriate, as heightened bleeding risk can offset ischemic benefit. This review reexamines the contemporary role of prasugrel, focusing on evidence supporting dose optimization, careful patient selection, and population‐specific considerations. Current evidence suggests that prasugrel remains a valuable antiplatelet option when tailored to individual ischemic and bleeding risks, particularly in patients undergoing complex percutaneous coronary intervention and selected Asian populations requiring individualized antiplatelet strategies.

## 1. Introduction

Cardiovascular disease remains the leading cause of morbidity and mortality worldwide [1], with atherosclerotic cardiovascular disease accounting for a substantial proportion of this global burden [2]. Atherosclerotic cardiovascular disease encompasses clinical conditions resulting from atherosclerosis of major arteries, including acute coronary syndrome (ACS), stroke, and transient ischemic attack [3]. ACS itself represents a continuum ranging from unstable angina to ST‐elevation myocardial infarction (STEMI) and nonST‐elevation myocardial infarction (NSTEMI) [4]. In the contemporary era of aggressive stable atherosclerotic cardiovascular disease management, antiplatelet therapy remains central to reducing ischemic events [5].

In patients with stable atherosclerotic cardiovascular disease, aspirin monotherapy remains the mainstay of antiplatelet therapy. By contrast, in those presenting with ACS or undergoing coronary stent implantation, dual antiplatelet therapy (DAPT) is essential to prevent recurrent ischemic events and stent thrombosis [6, 7]. The second agent in DAPT is typically chosen from the purinergic receptor subtype Y, G protein–coupled 12 (P2Y_12_) inhibitors, which block the platelet P2Y_12_ receptor to exert their antiplatelet effects. Among these, clopidogrel remains the most widely used, followed by prasugrel and ticagrelor, with selection guided by individual ischemic and bleeding risk, comorbidities, prior stroke or transient ischemic attack, and procedural factors associated with percutaneous coronary intervention (PCI) [4].

However, pharmacogenetic and ethnic differences can significantly influence antiplatelet response, particularly in Asian populations. Clopidogrel efficacy is frequently compromised by interindividual variability and nonresponsiveness, largely driven by genetic polymorphisms affecting drug metabolism, which are more prevalent in Asians and can result in suboptimal platelet inhibition and residual ischemic risk [8–10]. At the same time, these populations are more susceptible to bleeding complications, which may offset the ischemic benefits of potent P2Y_12_ inhibitors when standard Western dosing is applied [11]. Collectively, these factors emphasize that a one‐size‐fits‐all approach is inappropriate and highlight the need for dose individualization and population‐specific risk assessment.

Newer P2Y_12_ inhibitors, including prasugrel and ticagrelor, provide faster, more consistent platelet inhibition, and improved efficacy compared with clopidogrel [5]. Nevertheless, their broader adoption in Asian populations has been tempered by heightened bleeding risk and regulatory constraints. Despite these challenges, clinical evidence supports the careful use of prasugrel in selected Asian patients, emphasizing individualized dosing strategies, careful patient selection, and tailored therapeutic regimens that take into account ethnic variability and pharmacogenetic factors influencing drug response and bleeding risk. Against this backdrop, this review revisits the clinical relevance of prasugrel and explores its potential role in contemporary antiplatelet therapy, particularly in Asian populations. To achieve this aim, a literature search was conducted using PubMed, Scopus, and Web of Science databases for articles published up to 2025. The search terms used were “prasugrel”, “P2Y_12_ inhibitor”, “Asian”, “guidelines”, “pharmacokinetic”, “patients”, and “clinical trial.” Only clinical studies in patients with cardiovascular disease, including ACS, together with relevant systematic reviews, meta‐analyses, and clinical guidelines comparing or discussing prasugrel and other oral P2Y_12_ inhibitors, were included.

## 2. Pharmacology of Prasugrel

Prasugrel (marketed as Effient or Efient), a P2Y_12_ inhibitor developed by Daiichi Sankyo in collaboration with Eli Lilly, received approval in February 2009 for reducing atherothrombotic complications in patients with ACS who are treated with primary or delayed PCI [12]. It is available as oral prasugrel hydrochloride tablets in two strengths: 5 and 10 mg [13]. Prasugrel was designed to address the interindividual variability and inconsistent platelet inhibition associated with clopidogrel, and its potent antiplatelet efficacy initially generated considerable enthusiasm within the cardiology community.

**Figure 1:**
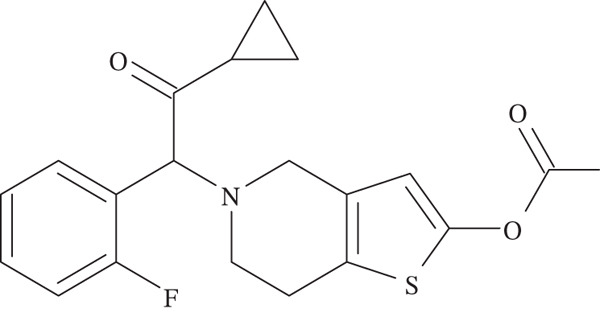
Chemical structure of prasugrel.

Prasugrel (Figure 1) is a thienopyridine‐class P2Y_12_ (ADP) receptor inhibitor, similar to clopidogrel. It exerts antiplatelet effects through irreversible inhibition of the platelet P2Y_12_ receptor, thereby suppressing ADP‐mediated platelet activation and aggregation [14]. Like clopidogrel, it is a prodrug; however, it requires hepatic activation through the cytochrome P450 isoenzymes CYP3A4 and CYP2B6, in contrast to clopidogrel, which is predominantly activated by CYP2C19 [5]. Unlike clopidogrel, prasugrel first undergoes rapid hydrolysis by esterases, resulting in more efficient formation of its active metabolite and substantially reduced interindividual variability in platelet inhibition, a phenomenon commonly observed with clopidogrel [15], potentially leading to better clinical outcomes in high‐risk patients. In clopidogrel, approximately 85% of the administered dose is hydrolyzed by esterases into inactive metabolites, with only a small fraction converted into the active metabolite [14]. This metabolic profile contributes to the lower functional bioavailability of clopidogrel compared with prasugrel. Comparative pharmacokinetic profiles of oral P2Y_12_ inhibitors are presented in Table 1.

**Table 1:** Comparative dosing characteristics, pharmacokinetic profiles and clinical considerations of commonly used P2Y_12_ inhibitors.

	Prasugrel	Clopidogrel	Ticagrelor
Route of administration	Oral	Oral	Oral
Frequency of administration	Once daily	Once daily	Twice daily
A prodrug	Yes	Yes	No
Functional bioavailability^†^ (%)	80	50	36
Onset of action (after loading dose) (hour)	0.5	2–6	0.5
Duration of action (day)	7–10	3–10	3–5
Withdrawal before surgery (day)	7	5	5
Metabolism (by major enzymes)	Activated by hepatic CYP3A4 and CYP2B6	Activated by hepatic CYP2C19	Hepatic CYP3A4
T_max_ (hour)	0.5	0.75	2–2.25
Genetic polymorphism	No	Yes	No
Half‐life^*ϕ*^ (hour)	0.5–1	0.5–1	6–12
Excretion	Urine (70%)	Urine (40%)	Urine (32%)
	Feces (30%)	Feces (60%)	Feces (68%)
Binding reversibility (P2Y_12_ receptor)	Irreversible	Irreversible	Reversible
Withdrawal before surgery ^∗^	~7 days	~5 days	~5 days

^†^Active metabolite for prasugrel and clopidogrel, and parent drug for ticagrelor. ^*ϕ*^active metabolite for prasugrel and clopidogrel, and parent drug/active metabolite for ticagrelor. ^∗^shortening may be considered if indicated by platelet function testing [5, 9, 14, 16–21].

Abbreviation: t_max_, time to maximum concentration.

## 3. Clinical Evidence Supporting Prasugrel′s Efficacy

Nearly two decades ago, despite its potent and consistent platelet inhibition, the clinical use of prasugrel was substantially limited by concerns over increased bleeding risk. In the Trial to Assess Improvement in Therapeutic Outcomes by Optimizing Platelet Inhibition With Prasugrel–Thrombolysis in Myocardial Infarction 38 (TRITON–TIMI 38), prasugrel administered as a 60‐mg loading dose before PCI, followed by 10 mg daily for up to 15 months, significantly reduced ischemic events, including stent thrombosis, in the overall ACS population; however, this benefit was accompanied by an increased risk of major bleeding, which frequently led to treatment discontinuation or switching [22]. On contrast, in the STEMI subgroup undergoing PCI, prasugrel conferred a net clinical benefit, with greater efficacy and no apparent excess in bleeding [23] (Table 2). These observations underscore the importance of appropriate patient selection when considering prasugrel therapy. Similarly, the Targeted Platelet Inhibition to Clarify the Optimal Strategy to Medically Manage Acute Coronary Syndromes (TRILOGY ACS) trial showed that prasugrel 10 mg did not significantly reduce cardiovascular events compared with clopidogrel in medically managed ACS patients [24]. Furthermore, A Comparison of Prasugrel at PCI or Time of Diagnosis of Non–ST‐Elevation Myocardial Infarction (ACCOAST) demonstrated that prasugrel pretreatment before coronary angiography in NSTEMI patients undergoing PCI conferred no ischemic benefit while increasing bleeding risk, supporting recommendations against its routine use in this setting [25]. On the basis of these findings, the 2015 European Society of Cardiology (ESC) guidelines contraindicate prasugrel in patients with prior stroke or transient ischemic attack, do not recommend prasugrel pretreatment, and advise its use only in patients with known coronary anatomy undergoing planned PCI [21].

**Table 2:** Major randomized controlled trials of prasugrel in cardiovascular disease in Western populations.

Trial	Region(s)	Subjects	Intervention	Major findings	Reference
TRITON–TIMI 38	North America, Europe, Australia/New Zealand, and Latin America	Moderate‐to‐high‐risk ACS patients with unstable angina or NSTEMI scheduled for PCI (*n* = 10,074)	Prasugrel 10 mg OD (LD 60 mg) vs. clopidogrel 75 mg OD (LD 300 mg) administered before PCI and continued for 15 months.	•Lower rates of ischemic events prasugrel.	[22]
				•Higher major and severe bleeding rates with prasugrel.	
				•Overall mortality did not differ between groups.	
TRITON–TIMI 38	North America, Europe, Australia/New Zealand, and Latin America	Patients with STEMI undergoing PCI (*n* = 3534)	Prasugrel 10 mg OD (LD 60 mg) vs. clopidogrel 75 mg (LD 300 mg) for 15 months.	•The rate of major adverse cardiovascular events was lower with prasugrel than with clopidogrel.	[23]
				•The rate of major bleeding with prasugrel was similar to that with clopidogrel.	
TRILOGY ACS	Europe, Mediterranean, Latin America, North America, Asia, Africa, Middle East, and Oceania	Patients with medically managed ACS (no PCI) (*n* = 7243)	Prasugrel 10 mg OD vs. clopidogrel 75 mg OD for 30 months.	•Prasugrel did not significantly reduce cardiovascular events compared with clopidogrel.	[24]
				•Bleeding risks were similar between the two drugs.	
ACCOAST	Europe, Middle East, and Canada	Patients with NSTEMI undergoing CABG (*n* = 314)	Prasugrel 10 mg OD (LD 60 mg at PCI) for 30 days vs. split LD 30 mg before angiography, then 30 mg at PCI, followed by daily 10 mg for 30 days.	•Prasugrel pretreatment increased major bleeding.	[25]
				•Prasugrel pretreatment did not impact ischemic outcomes.	
ISAR‐REACT 5	Germany and Italy	Patients with ACS undergoing PCI (*n* = 3377)	Prasugrel 10 mg OD (LD 60 mg) vs. ticagrelor 90 mg OD (LD 180 mg) administered before PCI and continued for ≥ 12 months together with aspirin.	•12‐month cumulative urgent revascularization rate higher with ticagrelor than prasugrel.	[26]
REDUCE‐MVI	Netherland and Spain	Patients with STEMI (*n* = 110)	Prasugrel 10 mg OD vs. ticagrelor 90 mg BD for 1 year.	•Platelet inhibition at 1 year was lower with ticagrelor.	[27]
				•No change in endothelial function with prasugrel, while improved with ticagrelor.	
NEO‐MINDSET	Brazil	Patients with ACS who undergone successful PCI (*n* = 3410)	Prasugrel (10 mg OD) or ticagrelor (90 mg BD) monotherapy vs. aspirin + ticagrelor or prasugrel for 12 months.	•P2Y_12_ inhibitor monotherapy was noninferior to dual antiplatelet therapy.	[28]
				•12‐month rates of death or ischemic events were similar between groups.	
FEATHER	United States, Ireland, Netherland, and Sweden	LBW and HBW patients with stable coronary artery disease taking aspirin (*n* = 72)	5 mg prasugrel vs. 10 mg prasugrel vs. 75 mg clopidogrel.	•Prasugrel 5 mg in LBW patients achieved platelet inhibition comparable to 10 mg in HBW patients.	[29]
				•Prasugrel 5 mg provided greater platelet inhibition than clopidogrel, with a comparable bleeding rate.	
GENERATIONS	United States, Ireland, Netherland, and Sweden	In aspirin‐treated very elderly and nonelderly patients with stable chronic coronary artery diseases (*n* = 155)	Prasugrel 5 mg vs. prasugrel 10 mg vs. clopidogrel 75 mg.	•In very elderly patients, prasugrel 5 mg was noninferior to 10 mg in nonelderly patients.	[30]
				•In very elderly patients, prasugrel 5 mg was more effective than clopidogrel.	

ACS, acute coronary syndrome; BD, twice daily; CABG, coronary artery bypass grafting; DAPT, dual antiplatelet therapy; HBW, high‐body‐weight; LBW, low‐body‐weight; LD, loading dose; NSTEMI, nonST‐segment elevation myocardial infarction; NSTE, non‐ST‐elevation; OD, once daily; P2Y_12_, purinergic receptor subtype Y, G protein–coupled 12; PCI, percutaneous coronary intervention; STEMI, ST‐segment–elevation myocardial infarction.

Recent clinical trials have demonstrated superior or comparable efficacy of prasugrel relative to ticagrelor. The Intracoronary Stenting and Antithrombotic Regimen: Rapid Early Action for Coronary Treatment 5 (ISAR‐REACT 5) trial evaluated patients with ACS undergoing PCI and showed that prasugrel 10 mg was superior to ticagrelor 90 mg in reducing the primary composite endpoint of death, myocardial infarction, or stroke, primarily through a reduction in recurrent myocardial infarction, without an increase in major [31]. A subsequent analysis of the trial further showed that prasugrel was associated with a lower incidence of urgent revascularization at 12 months compared with ticagrelor, with approximately half of urgent revascularization events involving remote coronary vessels [26]. In the Reducing Microvascular Injury Following Percutaneous Coronary Intervention (REDUCE‐MVI) trial, prasugrel 10 mg produced greater platelet inhibition than ticagrelor, highlighting the more consistent antiplatelet effect of prasugrel [27] (Table 2). A systematic review and meta‐analysis of 11 randomized trials reported that prasugrel had similar effects on the primary composite endpoint of major cardiovascular events (myocardial infarction, stroke, and cardiovascular death) at a weighted mean follow‐up of 11 months compared with ticagrelor [32]. Finally, in the Percutaneous Coronary Intervention Followed by Antiplatelet Monotherapy in the Setting of Acute Coronary Syndromes (NEO‐MINDSET) trial, prasugrel 10 mg monotherapy was not demonstrated to be noninferior to its combination with aspirin in terms of antiplatelet effect [28]. Collectively, these findings suggest that prasugrel may be considered for potent P2Y_12_ inhibition in carefully selected patients without contraindications.

The 2021 Asian Pacific Society of Cardiology (APSC) consensus does not specifically address prasugrel use in patients with chronic coronary syndrome but highlights the limited evidence available to guide antiplatelet therapy for multivessel coronary artery disease and de‐escalation strategies in Asian populations [33]. Since then, accumulating evidence from several clinical trials, including the ISAR‐REACT 5 trial [26, 31], has shaped contemporary recommendations.

The 2023 ESC ACS guidelines recommend prasugrel as the preferred P2Y_12_ inhibitor over ticagrelor in ACS patients undergoing PCI when not contraindicated, while emphasizing individualized bleeding risk assessment, including dose adjustment (standard dose: 10 mg daily; 5 mg in patients aged ≥ 75 years or with body weight < 60 kg) and consideration of de‐escalation strategies [4]. In parallel, the latest 2025 consensus from the American College of Cardiology (ACC), American Heart Association (AHA), American College of Emergency Physicians (ACEP), National Association of EMS Physicians (NAEMSP), and Society for Cardiovascular Angiography and Interventions (SCAI) recommends ticagrelor or prasugrel over clopidogrel in patients with ACS undergoing PCI, while advising against prasugrel use in patients with a history of stroke or transient ischemic attack [34]. Notably, neither guideline provides a formal recommendation for reduced‐dose prasugrel in selected populations.

The TRITON–TIMI 38 trial reported that patients with low body weight (< 60 kg) experienced an increased risk of bleeding when treated with the standard 10 mg dose of prasugrel [35]. These observations prompted the evaluation of lower‐dose prasugrel strategies in vulnerable populations. In the Comparison of Prasugrel and Clopidogrel in Low Body Weight Versus Higher Body Weight With Coronary Artery Disease (FEATHER) trial, platelet inhibition achieved with prasugrel 5 mg in low‐body‐weight patients was noninferior to that observed with prasugrel 10 mg in patients with higher body weight [29]. Advanced age is likewise associated with an increased bleeding risk compared with younger individuals [36], and in the Comparison of Prasugrel and Clopidogrel in Very Elderly and Non‐Elderly Patients With Stable Coronary Artery Disease (GENERATIONS) trial, prasugrel 5 mg in elderly patients (≥ 75 years) achieved antiplatelet effects comparable to those of the 10 mg dose while remaining superior to clopidogrel [30]. Collectively, these findings suggest that dose‐adjusted prasugrel regimens can preserve antiplatelet efficacy while potentially improving safety in vulnerable populations, particularly elderly and low‐body‐weight patients.

## 4. Efficacy of Reduced‐Dose Prasugrel in Asian Patients

Patients in the Asia–Pacific region exhibit a distinct benefit–risk profile for antiplatelet therapy compared with Western populations. In Asian populations, the risk of bleeding is generally higher, whereas the incidence of ischemic events is lower [37]. In addition, genetic polymorphisms affecting CYP2C19‐mediated bioactivation of clopidogrel are more prevalent among Asians, resulting in reduced antiplatelet effectiveness in a substantial proportion of patients [10]. Consequently, applying Western‐standard antiplatelet regimens in Asian patients may result in an unfavorable balance between ischemic protection and bleeding risk, highlighting the need for careful agent selection and dose optimization. These population‐specific factors have led to the use of lower‐dose prasugrel—or, in some countries, its discontinuation—due to safety concerns, bleeding risk, and regulatory restrictions [11], raising important questions about its current and future role in cardiovascular disease management in Asia.

Reflecting the challenges of antiplatelet therapy in Asian populations, several randomized clinical trials have recently been conducted in Japan, including Prasugrel and Clopidogrel in Japanese Patients With Ischemic Stroke (PRASTRO), Short and Optimal Duration of Dual Antiplatelet Therapy (STOPDAPT), and Acute Stroke or Transient Ischemic Attack Treated With Prasugrel (ACUTE‐PRAS) (Table 3). These studies evaluated lower doses of prasugrel (2.5–5 mg) and reported comparable incidences of ischemic and bleeding events relative to standard regimens [39–44]. In contrast, the Prasugrel Compared With Clopidogrel for Japanese Patients With ACS Undergoing PCI (PRASFIT‐ACS) trial demonstrated a reduction in ischemic events without an increase in bleeding risk in Japanese patients with ACS [38]. Collectively, these findings suggest that low‐dose prasugrel may be an effective antiplatelet strategy with a favorable or at least comparable safety profile in Asian populations, highlighting the importance of individualized dosing to balance ischemic protection and bleeding risk. Consistent with these observations, a network meta‐analysis of 13 randomized trials reported comparable rates of major adverse cardiovascular events between low‐dose prasugrel (3.75 mg) and clopidogrel [46]. Furthermore, a recent network meta‐analysis by De Filippo et al. [47] identified 12 months of DAPT with low‐dose prasugrel as a favorable strategy for reducing major adverse cardiovascular events and other ischemic outcomes, including myocardial infarction and stroke.

**Table 3:** Major randomized controlled trials of prasugrel in cardiovascular disease in Asian populations.

Study	Region	Subjects	Intervention	Major findings	Reference
PRASFIT‐ACS	Japan	Patients with ACS undergoing PCI were treated with aspirin (*n* = 1363)	Prasugrel 3.75 mg (LD 20 mg) vs. clopidogrel 75 mg (300 mg), both in combination with aspirin for 24–48 weeks.	•Lower incidence of ischemic events with prasugrel.	[38]
				•Lower risk of clinically serious bleeding with prasugrel.	
PRASTRO‐I, PRASTRO‐II and PRASTRO‐III	Japan	Patients with thrombotic stroke (*n* = 2688)	Prasugrel 3.75 mg OD vs. clopidogrel 75 mg OD for 4 and 24 weeks.	•PRU levels were lower with prasugrel than clopidogrel at 4 and 24 weeks.	[39]
STOPDAPT‐3	Japan	Diabetic patients with ACS or high bleeding risk undergoing PCI (*n* = 5966)	Prasugrel 3.75 mg OD (LD 20 mg) vs. prasugrel 3.75 mg OD (LD 20 mg) and aspirin 81–100 mg OD (LD 162–200 mg) for a month.	•Similar coprimary bleeding and cardiovascular events with prasugrel alone vs. DAPT, regardless of diabetes.	[40]
ACUTE‐PRAS	Japan	Patients with acute large artery atherosclerosis or high‐risk TIA (*n* = 176)	Prasugrel 3.75 or 2.5 mg OD without loading vs. clopidogrel 75 or 50 mg OD with loading.	•Prasugrel showed significantly lower Day 5 PRU in the overall, EM, and IM groups, with a similar trend in PMs.	[41]
				•Prevalence of new infarct lesions, adverse events, and bleeding were comparable between groups.	
STOPDAPT‐3	Japan	Patients with ACS or high bleeding risk on OAC undergoing PCI (*n* = 5966)	Prasugrel 3.75 mg OD (LD 20 mg) vs. prasugrel 3.75 mg OD (LD 20 mg) + aspirin 81–100 mg OD (LD 162–200 mg) for a month.	Major bleeding incidence was similar with prasugrel alone and DAPT.	[42]
PRASTRO‐I	Japan	Patients with ischemic stroke (*n* = 3753)	Prasugrel 3.75 mg OD vs. clopidogrel 75 mg OD for 96 weeks.	•Similar event rates between groups across all stroke subtypes.	[43]
PRASTRO‐III	Japan	Patients with ischemic stroke (*n* = 230)	Prasugrel 3.75 mg OD vs. clopidogrel 75 or 50 mg OD for 24–48 week	Bleeding incidence was also similar across all subtypes. A risk reduction of 5% with prasugrel vs. clopidogrel.	[44]
—	Japan	Patients with noncardioembolic stroke (*n* = 63)	Prasugrel 2.5 mg, 5 mg, or 7.5 mg vs. clopidogrel 75 mg OD for 14 days.	Platelet inhibition was dose‐dependently higher with prasugrel.	[45]

Abbreviations: ACS, acute coronary syndrome; DAPT, dual antiplatelet therapy; EM, extensive metabolizers; IM, intermediate metabolizers; LD, loading dose; OAC, oral anticoagulant; OD, once daily; PCI, percutaneous coronary intervention; PM, poor metabolizers; PRU, platelet reaction unit; TIA, transient ischemic attack.

Although prasugrel is generally contraindicated in patients with prior stroke or transient ischemic attack based on TRITON–TIMI 38, selected Japanese trials evaluated reduced‐dose prasugrel in patients with cerebrovascular disease under strict inclusion criteria [41, 43–45]. These studies were conducted within a distinct regulatory framework that approved lower prasugrel doses to address bleeding susceptibility and were designed to assess safety and pharmacodynamic efficacy rather than to revise established contraindications. Accordingly, their findings should not be extrapolated to standard‐dose prasugrel or broader populations without further confirmatory evidence. Nevertheless, these data suggest that reduced‐dose prasugrel is pharmacodynamically active and may be feasible in carefully selected Asian populations, highlighting the potential for individualized dosing strategies while underscoring the need for further prospective studies to confirm safety and efficacy.

A trial by Yamaguchi et al. [45] compared three doses of prasugrel (2.5, 5, and 7.5 mg) with clopidogrel in Japanese patients with noncardioembolic stroke, demonstrating dose‐dependent platelet inhibition, with substantial antiplatelet effects observed even at the lowest prasugrel dose. Extending these pharmacodynamic observations to clinical settings, real‐world evidence from a Taiwanese registry showed that low‐dose prasugrel (3.75 mg) provided effective ischemic protection with a low risk of bleeding in patients with ACS [48]. Consistently, analyses from two South Korean registries reported that low‐dose prasugrel was associated with reduced major adverse cardiac and cerebrovascular events and greater effectiveness compared with clopidogrel, while maintaining a comparable bleeding risk in elderly and low‐weight ACS patients [49]. Collectively, these findings suggest that lower‐dose prasugrel may offer a favorable balance between antiplatelet potency and clinical safety in Asian patients, supporting dose individualization antiplatelet therapy in routine practice.

One method of assessing platelet inhibition is the quantitative measurement of platelet reactivity, expressed as platelet reaction units (PRU). In the ACUTE‐PRAS trial, the effects of low‐dose prasugrel (2.5 or 3.75 mg) on platelet reactivity were compared with those of clopidogrel in Japanese patients with acute large‐artery atherosclerosis or high‐risk transient ischemic attack. Prasugrel achieved significantly lower PRU values at Day 5 than clopidogrel in the overall population (136.5 vs. 159.4), irrespective of metabolizer status. Furthermore, among patients with high on‐treatment platelet reactivity, the proportion was lower in those receiving prasugrel [39]. Consistent results were also reported at 4 and 24 weeks in the Japanese PRASTRO‐I, PRASTRO‐II, and PRASTRO‐III trials with prasugrel 3.75 mg [39]. Given that higher PRU values are associated with an increased risk of ischemic events [50], these results indicate that low‐dose prasugrel provides more effective platelet inhibition and may be associated with improved ischemic risk reduction while maintaining an acceptable safety profile.

Few recent studies have evaluated low‐dose prasugrel‐based strategies as a form of de‐escalation, possibly because low‐dose prasugrel has demonstrated comparable or lower bleeding risk than ticagrelor or clopidogrel. In the Chronic Phase of Post‐PCI (CHAPERON) study, a prospective cohort study of Japanese patients with coronary artery disease undergoing PCI, step‐down de‐escalation from prasugrel 3.75 to 2.5 mg maintained low PRU values, suggesting sustained platelet inhibition. Notably, switching from clopidogrel 75 mg to prasugrel 2.5 mg resulted in significantly reduced PRU values, indicating greater platelet inhibition with prasugrel compared with standard‐dose clopidogrel [51]. Collectively, these findings suggest that low‐dose prasugrel may have potential roles both as a dose‐reduction strategy for bleeding risk mitigation and as an alternative maintenance option in selected patients; however, further studies are needed to define its clinical efficacy and safety.

Additional evidence is provided by a recent network meta‐analysis by De Filippo et al. [47], which evaluated DAPT de‐escalation strategies, predominantly involving early switching from potent P2Y_12_ inhibitors (prasugrel or ticagrelor) to clopidogrel. De‐escalation was associated with reduced bleeding without an increase in cardiovascular death or ischemic outcomes, supporting its role as a bleeding‐avoidance strategy after PCI. In the same analysis, low‐dose prasugrel‐based DAPT was associated with a lower risk of overall bleeding compared with standard‐dose prasugrel–based therapy.

Randomized clinical evidence on prasugrel in Asian populations is derived almost exclusively from Japanese cohorts, in whom reduced‐dose regimens were adopted to mitigate bleeding risk. As emphasized in the 2020 APSC consensus recommendations, Asian patients are markedly under‐represented in pivotal DAPT trials, and Western guideline recommendations may not be directly applicable to Asia–Pacific populations because of differences in genetic background, body size, comorbidity profiles, and bleeding susceptibility [11]. Although Japanese prasugrel trials provide important proof of concept for dose adjustment and individualized antiplatelet therapy in East Asian patients, they do not fully capture the heterogeneity of the broader Asian population.

The 2021 APSC further emphasized that Asia comprises highly diverse ethnic and clinical subgroups, and extrapolation of Japanese prasugrel data to other Asian populations should be approached with caution [33]. Variations in CYP2C19 polymorphisms, pharmacokinetics, baseline ischemic and bleeding risks, and clinical practice patterns across East, Southeast, and South Asia may substantially influence the benefit–risk profile of potent P2Y_12_ inhibitors. Although reduced‐dose prasugrel appears effective and safe in Japanese patients, current evidence is insufficient to assume that the same regimen is optimal across all Asian populations. Therefore, although reduced‐dose prasugrel may be considered in East Asian patients where data exist, its use in other Asian populations should be individualized based on patient characteristics and clinical judgment, highlighting the need for region‐specific randomized trials and real‐world evidence.

Nevertheless, reduced‐dose prasugrel (e.g., 3.75 mg as evaluated in clinical trials in Japan) is not universally approved and remains largely limited to specific regions such as Japan, South Korea, and Taiwan. In many other Asian countries, its use is constrained by regulatory status and drug availability, and may involve off‐label prescribing or access to imported formulations. In some Southeast Asian settings, including Malaysia, prasugrel is not routinely used in clinical practice and may be considered only on a case‐by‐case basis according to clinician judgment and individual patient risk profiles.

## 5. Summary and Future Directions

Prasugrel provides more potent and consistent platelet inhibition than clopidogrel, and may serve as an alternative to ticagrelor in patients with ticagrelor‐associated dyspnea, intolerance, or concerns regarding twice‐daily dosing. From a clinical perspective, it may be considered in selected patients with high ischemic risk and acceptable bleeding risk, particularly those with ACS undergoing primary PCI, extensive coronary artery disease, prior stent thrombosis, or high on‐treatment platelet reactivity while receiving clopidogrel. In these settings, prasugrel—at standard or reduced doses according to bleeding risk—may improve ischemic protection through more consistent platelet inhibition than clopidogrel.

However, its higher bleeding risk—particularly in Asian populations—highlights that a one‐size‐fits‐all approach is inappropriate and underscores the importance of careful patient selection and individualized dosing. Although reduced‐dose prasugrel strategies have demonstrated favorable efficacy–safety profiles in several Asian studies, the available evidence remains largely derived from limited regional data, constraining broader generalizability across Asia. This emphasizes the need for region‐specific randomized trials and personalized antiplatelet strategies guided by genetic or platelet function testing in patients with heightened bleeding susceptibility. Ultimately, optimal use of prasugrel requires balancing ischemic protection against bleeding risk, supported by population‐specific evidence and real‐world data to inform individualized therapy in contemporary cardiovascular care.

De‐escalation from prasugrel or switching to a less potent P2Y_12_ inhibitor should be considered in patients with bleeding complications, stable postACS status, or need for oral anticoagulation or invasive procedures. Dose reduction or switching may also be warranted with increasing bleeding risk due to age, low body weight, or comorbidities. Conversely, prasugrel should be avoided or used cautiously in elderly patients, those with low body weight, or those with prior stroke or transient ischemic attack, where reduced‐dose or alternative P2Y_12_ inhibitors may be preferred.

Future research should prioritize individualized antiplatelet strategies, including the integration of platelet function testing and genetic testing to guide P2Y_12_ inhibitor selection, dosing, and de‐escalation approaches. In addition, adequately powered head‐to‐head trials comparing reduced‐dose prasugrel with ticagrelor across diverse Asian populations are needed to better define optimal treatment strategies. Further evaluation and development of lower‐strength prasugrel formulations may also improve the balance between ischemic protection and bleeding risk in populations with heightened bleeding susceptibility. Ultimately, population‐specific treatment approaches supported by real‐world registry data and region‐specific randomized trials will be essential for optimizing the role of prasugrel in contemporary cardiovascular care.

**Clinical Take-Home Messages**

• Prasugrel provides more potent and consistent platelet inhibition than clopidogrel and may reduce ischemic events in selected high‐risk ACS patients.
• Prasugrel may be an alternative to ticagrelor in selected patients with ticagrelor‐induced dyspnea, intolerance, or adherence concerns, with careful patient selection essential.
• Bleeding risk—especially in Asian populations—necessitates individualized prasugrel dosing and de‐escalation strategies.
• Evidence on optimal prasugrel dosing and de‐escalation strategies across diverse Asian populations remains limited and warrants further investigation.

## Author Contributions

Yusof Kamisah and Hamat Hamdi Che Hassan contributed equally to the conception and design of the article, drafting, critical revision, and final approval.

## Funding

This review article is funded by the Fundamental Grant of Faculty of Medicine, Universiti Kebangsaan Malaysia, FF‐2024‐241.

## Disclosure

The authors declare that generative AI (ChatGPT) was utilized solely for sentence revision in the preparation of this manuscript.

## Conflicts of Interest

The authors declare no conflicts of interest.

## Data Availability

Data sharing is not applicable to this article, as no new data were generated or analyzed.
